# Crowdfunding Curriculum Design Based on Outcome-Based Education

**DOI:** 10.3389/fpsyg.2022.845012

**Published:** 2022-04-14

**Authors:** Yenchun Jim Wu, Chih-Hung Yuan

**Affiliations:** ^1^Graduate Institute of Global Business and Strategy, National Taiwan Normal University, Taipei, Taiwan; ^2^College of Humanities and Arts, National Taipei University of Education, Taipei, Taiwan; ^3^School of Economics and Commerce, University of Electronic Science and Technology of China, Zhongshan Institute, Zhongshan, China

**Keywords:** curriculum design, outcome-based education (OBE), crowdfunding, entrepreneurship, entrepreneurial ventures

## Abstract

Entrepreneurship has flourished in recent years; however, since education on how to raise funds has received little attention from scholars, obtaining funds remains a difficult task. The development of crowdfunding has provided new opportunities to entrepreneurs, thus solving the funding, marketing, and distribution problems they previously faced. The main purpose of this study is to organize crowdfunding literature and to develop a crowdfunding curriculum grounded on output-based education. Students are asked to develop a product and a crowdfunding plan within the span of one semester. This study explains the teaching content separately from the crowdfunding plan competition, course elements, and timetable.

## Introduction

Crowdfunding as a practice has flourished in recent years and has become a new way for entrepreneurial ventures, or even individuals, to obtain venture capital (Kazakeviciute et al., 2016). It is an Internet-based financial model which supports entrepreneurs’ efforts in raising funds for their projects from a relatively large number of individuals, rather than *via* traditional financial intermediaries (Tuo et al., 2019). The market size of global crowdfunding in 2019 was estimated to be approximately US $30 billion; it is further estimated that, from 2021 to 2026, the compound annual growth rate will exceed 16% (Mordor Intelligence., 2021). The same report indicated that in 2019, 6,445,080 fundraising campaigns were held globally.

Some scholars have focused their fundraising research on key success factors (Simon et al., 2019; Zhang et al., 2020; Wang et al., 2021), investment motivations (Liang et al., 2019; Wang et al., 2022), delivery performance (Tuo et al., 2019), and funding types (Figueroa-Armijos and Berns, 2021). However, crowdfunding education have received less attention from researchers than entrepreneurship education (Vealey and Gerding, 2016; Luo et al., 2018; Ruget, 2019; Shneor and Flåten, 2020).

Many universities and colleges currently offer diverse programs in entrepreneurship. When students identify unmet needs, they develop creative problem-solving products or services to discover potential business opportunities. However, the enthusiasm of individuals or teams alone can only transform ideas into prototypes; if there is a lack of funding, these ideas cannot be commercialized. Therefore, due to limitations in knowledge, resources, and experiences, many new entrepreneurs face higher failure rates than their older counterparts (Stamboulis and Barlas, 2014). The crowdfunding platform is an emerging model of Internet finance, providing entrepreneurs with new opportunities to solve the problems regarding funding, marketing, and distribution. Before commercializing their ideas, entrepreneurs thus have a chance to obtain funding during the ideation, creation, or post-production stages.

Through crowdfunding, founders can realize diverse goals, including fundraising, demand testing, and marketing. Funders are able to propose existing ideas to seek financial support from backers (Copeland, 2015). They can observe users’ responses directly on the Internet, interact with consumers, and compete to test the market without distributing a large number of goods at once. On crowdfunding platforms, backers are characterized by diversity in backgrounds, high levels of education, and high degrees of professionalism. If the backers of the platform are not interested in a particular project—and this results in a deficiency of demand—there will be no need for founders to invest additional funds or efforts. Crowdfunding has also been used for marketing purposes, to generate demand for new projects in their early stages. Backers can be used to test consumers’ responses to products and may help entrepreneurs identify the market prospects facing their products or projects. This can also cultivate early users’ sense of belonging to products or projects. It is particularly important for industries in which projects seek to create an ecosystem for free products (Mollick, 2014). Hence, how the founder designs the crowdfunding campaign becomes very important. Crowdfunding campaigns that lack academic consideration do not necessarily reduce the writing quality on the platform (Copeland, 2015). Crowdfunding curriculums, however, can help students develop a better and systematic understanding of how to raise funds, demonstrate demands, and engage in marketing. Therefore, crowdfunding education can help students be ready for the evolving nature of contemporary entrepreneurial work.

The curriculum of this paper adopts the principles of outcome-based education (OBE). Students are asked to develop a product and a crowdfunding plan over one semester. OBE originated out of behavioral learning theory, with a focus on observable behaviors. It has been defined as “clearly focusing and organizing everything in an educational system around what is essential for all students to be able to do successfully at the end of their learning experience” (Spady, 1994). The concept of OBE provides the structure and system for a learning outcome-driven curriculum; the role of student-centered education in achieving the objectives and requirements of the curriculum is clear. Making a direct connection between curriculum, objectives, and requirements is a critical first step.

The main objective of a course based on such a curriculum would be to provide learners with practical experience so that they can understand the work required to initiate crowdfunding campaigns—this includes developing new products, writing project descriptions, making videos, and engaging in fundraising on crowdfunding platforms. The students will have a preliminary understanding of how to launch crowdfunding campaigns before raising funds online. The literature on online crowdfunding is growing rapidly, and scholars are devoted to studying and explaining the factors in the success of such endeavors. For example, Tafesse (2021) referred to theoretical insights from the advertising communication literature and employed a dataset of more than 8,000 reward-based crowdfunding campaigns to test the effects of communication strategies on the campaigns. Eiteneyer et al. (2019) claimed that inviting clients to participate in the innovative activities of entrepreneurial ventures can significantly increase the success rate of crowdfunding for new products. This study will extract the main research topics from journal articles that will be regarded as potential teaching content.

Crowdfunding campaigns can be generally classified into four types: donation-based, lending-based, equity-based, and reward-based (Figueroa-Armijos and Berns, 2021). Reward-based crowdfunding is most commonly used, accounting for 74% of the industry share in 2019 (Valuates Reports, 2019). Therefore, based on OBE, the design of the course focuses on reward-based crowdfunding campaigns. The rest of the article is organized as follows. Section “Crowdfunding Course Overview” explains outcome-based competition activities and teaching objectives. Section “Content of the Crowdfunding Course” introduces the development of the crowdfunding curriculum and its content. Finally, section “Conclusion” proposes some pedagogical implications and concludes the study.

## Crowdfunding Course Overview

### Crowdfunding Plan Competition

This course integrates the technology commercialization model of OBE and Jolly (1997) to develop students’ competencies in product development and crowdfunding. Outcomes in the OBE concept are not grades, but competencies that students have at the end of their learning journey (Spady, 1982). These competencies include knowledge and understanding (cognition), practical skills (skills), attitudes and values (affection), and individual behavior (Gullickson, 2003). The course project results can be divided into multiple sub-tasks, and the sub-tasks must be related to each other and show the staged output results. This task structure helps students know what they need to do and stay focused and understand why the task is being done. As students try to understand the task, they design solutions and turn them into action plans (Rao, 2020). An important difference between OBE and traditional education is teaching feedback (Ansari and Usmani, 2018). Feedback is a mechanism that drives student progress, leading to better and more effective learning experiences (Wu et al., 2012). Jolly (1997) believes that innovation commercialization refers to a series of processes from the generation of innovative technology to the entry of products, embodying the new technology into the market. He analyzes the innovation value chain from the perspective of product life cycle, and proposes five stages of commercialization, including imagination, cultivation, physical display, promotion, and sustainability. Only innovations that can improve corporate profitability are successful innovations, which depend not only on the technological R&D capabilities of the company but also on the subsequent excellent commercialization process.

**FIGURE 1:**
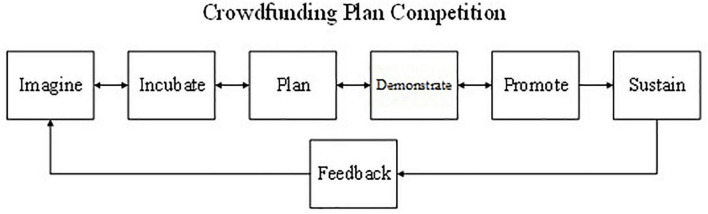
Crowdfunding plan competition. Adapted from Evans et al. (2007) and Jolly (1997).

Based on OBE, this course slightly modifies the technology commercialization model, adding planning and feedback. Students who enrolled in this course were asked to develop their products and crowdfunding plans through three rounds of competitions. In each stage, clear learning objectives and outcomes were arranged. Only by allowing students to constantly act and reflect in the process of practice will they obtain results and abilities. The procedures used in the crowdfunding plan competition are shown in Figure 1. We provided opportunities for product development before the stage in which formal crowdfunding plans were prepared, allowing learners to complete their proposals step-by-step. The following points will explain the procedures:

1. Imagining: This is the process of value generation and the beginning of the creative process. It involves activities such as identifying unmet needs in the market or the deficiencies of existing products and innovatively proposing patent ideas or new products or services to meet these needs.
2. Incubating: A wide range of stakeholders work together to prove the feasibility of the idea. At this stage, concepts and ideas form a prototype, which is then continuously improved and commercialized.
3. Planning: The course module requires carrying out different types of planning for products, descriptions, and videos, among others. The main objective of this stage is to use innovation and creativity in planning.
4. Demonstrating: In class, each group demonstrates its plan and innovation idea. The crowdfunding plan is continuously refined through the three rounds of elevator pitches until a good plan is formulated. This stage aims to cultivate students’ teamwork, integration, improvement, and expression competencies.
5. Promoting: Through enthusiasm and appropriate preparation, each team promotes its plan to the reviewers or even to crowdfunding platforms and potential sponsors later. The main objective of this stage is to quickly convey to the reviewer or sponsor, the advantages of the product and evoke their interest.
6. Sustaining: The most important purpose of crowdfunding is to raise funds to make products. The products must be produced and delivered to the sponsor; otherwise, the funds will be refunded. Founders should continuously carry out incremental research and development to improve existing technologies and achieve sustainability.

### Teaching Objectives

The components of the crowdfunding education program are shown in Table 1. We have determined a philosophical teaching approach that can guide the “pedagogy, teaching, and learning process” and the roles of participants in the course (Kazakeviciute et al., 2016). This study employed the components of a crowdfunding education program developed by Maritz and Brown (2013), including the objectives, content, pedagogy, and audience. The details are explained as follows:

**TABLE 1:** The components of a crowdfunding education program.

Dimension	Description
Objectives	Acquiring knowledge that is closely related to crowdfunding Learning how to move from idea, to product, to crowdfunding Understanding the strategies, operational methods, risks, and benefits of crowdfunding Understanding the management of network communities Learning from cross-disciplinary and practical experience
Audience	Full-time undergraduate and graduate students
Pedagogy	75% lectures and 25% self-directed learning
Content	Basic principles of crowdfunding, product development, innovation, and marketing
Assessment	Elevator pitch 75% and term paper 25%. Rating for elevator pitch according to the teams’ entrepreneurial proposals and the demonstration of crowdfunding campaigns. The final report is graded by the instructor.
Outcomes	Knowledge about product development and crowdfunding behaviors are usually derived from group work on interesting real-world projects.

*Based on Maritz and Brown (2013).*

1. Objectives: Teaching objectives refer to the expectations to be met during the teaching process (Maritz, 2017). They are also the basis for teachers’ choices of pedagogy, content, assessment, and outcomes. The objectives of the present course are: (1) to learn how to transform ideas into products and demonstrate them in crowdfunding campaigns; and (2) to use marketing plans and network communities to make crowdfunding plans successful.
2. Audience: The learners of this course are not required to have previously enrolled in Entrepreneurial Management, and all full-time students can enroll in this course. We encourage learners to form interdisciplinary teams, and if possible, to not limit the members of the teams to students who enroll in this course. The team instructors are not limited to lecturers, and the teams can invite teachers from other disciplines or business experts. Such diversity can provide greater opportunities for mutual learning (Kazakeviciute et al., 2016).
3. Pedagogy: Lectures account for 75% of the curriculum, including basic concepts, case studies, crowdfunding process, and crowdfunding plan demonstration. Self-directed learning accounts for 25%, covering new product development, factory contacting, and crowdfunding planning. Lectures, case studies, presentations, discussions, and elevator pitches are the main teaching methods.
4. Content: This course focuses on developing entrepreneurial knowledge, skills, and competencies through a greater emphasis on searching for market opportunities and commercializing ideas than on theory-oriented learning. The teaching content is divided into four modules: development of entrepreneurial projects, project descriptions for crowdfunding, video production, and entrepreneurial sustainability. These teaching contents are organized by relevant literature.
5. Assessment: Course grades consist of three rounds of team lift competitions (75%) and individual final reports (25%). In the competition, each round accounts for 25%, and the points come from two evaluation systems. The first assesses thelearners’ coursework delivery and content (10%), and the second is the peer rating of the elevator pitches (15%). In the first system, the instructor assesses the white paper delivered by the learners, which includes the final drafts of the product proposal, project descriptions for crowdfunding, video design, and the crowdfunding proposal. In the second system, students rate the elevator pitches of other groups. The instructor ranks the ratings after averaging them and assigns scores across different ranges of ratings. This design allows learners to not only understand the advantages of other groups’ approaches, but also improve their own approaches. The personal final report consists of the practical training process record (15%) and learning experience (10%) and is scored by the instructor. The record of the practical training process includes the purpose, time arrangement, content, difficulties encountered, and solutions. The learning experience must be combined with professional knowledge to summarize the feelings, experiences, gains, opinions, and suggestions during the training period
6. Outcomes: Three rounds of elevator pitches are to be presented during lectures. Each round focuses on coherent modules with different content. Students will be asked to formulate a crowdfunding plan through simple group exercises and demonstrations.

## Content of the Crowdfunding Course

### Development of Teaching Content

Previous studies have discussed how to be successful *via* crowdfunding from a number of different perspectives such as new product development (Chan et al., 2021; Roma et al., 2021; Wu and Chen, 2021a), crowdfunding project design (Moradi and Badrinarayanan, 2021; Felipe et al., 2022), multimedia and social media (Datta et al., 2019), and financial sustainability (Gamble et al., 2017). This study organizes the above into modules, contents, and references (Table 2).

**TABLE 2:** Crowdfunding curriculum development.

Module	Content	References
General information	Crowdfunding platform	André et al., 2017
	Entrepreneurship team	Gafni et al., 2019
New product development	Opportunity recognition	Eiteneyer et al., 2019
	Innovation orientation	Datta et al., 2019; Sahaym et al., 2021
	New product development	Roma et al., 2021; Sahaym et al., 2021; Tafesse, 2021
	Sustainability	Chan et al., 2021
	Product cost	Liu et al., 2021
Crowdfunding project design	Project descriptions	Burtch et al., 2013; Yuan et al., 2016; Moradi and Badrinarayanan, 2021; Tafesse, 2021
	Price and rewards	Liu et al., 2021; Felipe et al., 2022
	New project revision	Cornelius and Gokpinar, 2020
	Funding target	Liu et al., 2021
Multimedia and social media	Photos	Tafesse, 2021
	Videos	Gafni et al., 2019; Tafesse, 2021
	Social media	Datta et al., 2019; Sahaym et al., 2021
	Customer	Eiteneyer et al., 2019; Cornelius and Gokpinar, 2020
	Communication	Chan et al., 2021
Financial sustainability	Financial sustainability	Gamble et al., 2017
	Product delivery	Tuo et al., 2019

### Teaching Content

Outcome-based education divides teaching activities based on two categories: teachers and students. The teachers are expected to achieve training objectives that target five competencies: self-directed learning, hands-on experience, team communication and cooperation, problem analyzing and solving, and innovation. Meanwhile, learners are expected to meet project requirements, complete the prescribed assignments, and attain a certain level of innovative achievement. Based on Table 2, the crowdfunding curriculum is divided into four stages, which are presented in the course timetable (Table 3) for the semester.

**TABLE 3:** Timeline of the course.

Dates	Instructor	Students	Deliverables
Week 1	Explanation of crowdfunding campaigns, group competition, and how to recognize market opportunities	Formation of teams (interdisciplinary) and observation of market demands	Crowdfunding campaign competition
Week 2	Introduction to entrepreneurial thinking and ways of finding solutions	Visualization of a solution based on market demands	Draft proposals of the products
Week 3	Explanation of prototype incubation	Designing of prototypes and initiation of search for factories to create samples	Final drafts of product proposals
Week 4	No class. Meet with teams facing difficulties and help them solve their problems.	Self-directed learning and preparing for the first round of elevator pitches	
Week 5	Explanation of the assessment rubric. Offer suggestions and make assessments after the competition.	Presentation regarding market demand and proposal regarding products or services. Delivery of peer rating and feedback.	First round of elevator pitches
Week 6	Introduction to crowdfunding and its working mechanisms	Learners start to plan their crowdfunding proposal	Outline of crowdfunding project descriptions
Week 7	Case studies on marketing plans and crowdfunding	Students conduct case studies on crowdfunding	Drafts of crowdfunding project descriptions
Week 8	Successful professionals to be invited to share their crowdfunding experiences	Learners start to design the webpage on crowdfunding platforms and confirm the production cost with factories	Final drafts of crowdfunding project descriptions
Week 9	No class. Meet with teams facing difficulties and help them solve their problems.	Self-directed learning. Prepare for the second round of elevator pitches	
Week 10	Explanation of the assessment rubric for the second round. Offer suggestions and make assessments after the competition.	Students present their project descriptions for crowdfunding. Peer rating and feedback delivered.	Second round of elevator pitches
Week 11	Introduction to crowdfunding videos and photo productions	Learners start to design the videos and photos for crowdfunding	Draft design of fundraising videos
Week 12	Explanation of social media management and advertisement	Website and social media management. Confirmation of the production schedule with factories.	Final drafts of fundraising videos
Week 13	No class. Meet with the teams facing difficulties and help them solve their problems.	Self-directed learning. Integrating products, photos, videos, pitches, and reward plans with social network sites.	Drafts of crowdfunding proposals
Week 14	No class. Meet with the teams facing difficulties and help them solve their problems.	Self-directed learning. Preparation for the third round of elevator pitches.	Final drafts of crowdfunding proposals
Week 15	Explanation of the assessment rubric for the third round. Offer suggestions and make assessments after the competition.	Students present the final crowdfunding campaign. Peer rating and feedback delivered.	Third round of elevator pitches
Week 16	Explanation of how to maintain the sustainability of products. Encourage excellent teams to raise funds on crowdfunding platforms. Course wrap-up.	Reflection and feedback on this course	Term papers

During the first stage (Weeks 1–5), the instructor introduces the course content, explains the semester’s three-round group competition, and asks students to shift from a merely “thinking”-based learning approach to “practical” learning. Then, the instructor explains how to recognize market opportunities and imagine and design services or products in an orderly manner. Students form teams and observe market demands. Then, based on imaging and design, they propose solutions. Sustainability and innovation are important considerations when designing a product (Wu and Chen, 2021b). Entrepreneurs who are sustainability- and innovation-oriented can be more successful at crowdfunding (Chan et al., 2021). In Week 5, each team needs to, quickly and briefly, explain their business ideas, novel factors, and key differentiations with competitors. The length of the presentation is limited to 1 min, with a maximum of three slides. Other teams will rate and give feedback on the presentation. After completing this module, learners will be able to design product or service prototypes.

The target customers of a product can be identified through crowdfunding platforms. During the second stage (Weeks 6–10), the lecturer explains the key components and sponsorship aspects of crowdfunding campaigns. By discussing the product description examples in terms of different qualities, students will understand what makes information appealing and develop effective communications for a crowdfunding campaign. Sponsorship is the act of participating financially in a project where the backer provides a certain amount of funding to the proposer and receives substantial rewards related to the project. Student teams work on a crowdfunding proposal, determining the target amount, product description, and plan to reward consumers. When setting the target amount for crowdfunding, students must consider the cost of the reward plan. Project title, project content, team introduction, commitment, and risks are essential components of project descriptions. Factors such as frequent updates can contribute to successful outcomes, whereas spelling errors will reduce the success rate (Mollick, 2014). A reward plan consists of one or more reward projects. The ingenious arrangement of the components and pricing of reward plans can help backers make choices. In Week 10, each team needs to introduce their project descriptions. The length of the presentation is limited to 3 min, with a maximum of six slides. Other teams give their ratings and feedback on the presentation. When this module is over, learners will be able to design a plan that either resonates with the target audience or creates a perception of need.

In the third stage (Week 11–15), the lecturer explains the essential elements of videos and the management of social media, addressing the question of how to make a project video that can clearly convey the ideas to sponsors; this is done by discussing examples of videos with different qualities that will help students understand the process behind good videos. Social networking has an important role in funding entrepreneurial ventures, as crowdfunding is, by nature, a highly socialized behavior. A study on the relationship between crowdfunding sponsors and proposers revealed that the number of friends on online social networks positively correlated with success (Mollick, 2014). Therefore, before the project is opened for online fundraising, it is best to cultivate a group of fans who are concerned about the project. Based on their products and potential sponsors, learners start to plan the themes and scripts of the video, including purposes and plots. A crowdfunding video usually focuses on a specific “purpose,” which serves as the core principle for developing all the scenes and scripts. In addition, for proposers, a fan club is a medium for engaging with the public, directly connecting the project and the crowd. Before the project goes online, it is recommended to draft 1–2 months of posts and appropriately plan their themes and contents. In Week 15, each team should introduce their crowdfunding plan within 3 min. Other teams provide ratings and feedback on their presentations. After completing this module, students will be able to develop a crowdfunding proposal to attract sponsors.

In the final stage (Week 16), the lecturer explains how to maintain financial sustainability and product delivery performance. A major advantage of crowdfunding is the feedback received from backers, which can strengthen the two-way communication between founders and those providing support (monetary and otherwise). Continuous improvement in products or crowdfunding campaigns is required to realize financial sustainability. Finally, students are encouraged to use their plans to raise funds on crowdfunding platforms after the course. The course is subsequently concluded.

### Outcomes

Through this course, it is hoped that the student teams can explore market opportunities and innovatively develop a new product or service. It is difficult to develop a new product or service in a short amount of time. However, the lecturer can provide examples to illustrate how entrepreneurs put forward entrepreneurial plans to help students think and develop. For example, yak is a special local livestock originating from the Qinghai-Tibet Plateau. An entrepreneur, Danma, found that the vitamin A and fat contained in yak milk can moisturize skin and promote epidermal metabolism. With this understanding, she made handmade soaps using yak milk without additives, going on to sell the product across the world. Another social enterprise, NORLHA, found that Tibetans seems to have neglected other uses of yak wool, which had the potential to produce extreme wealth and which had accompanied Tibetans for over a thousand years. After cleaning and depilation, yak wool is woven into textiles, lending surprising softness, warmth retention, and durability. These exquisite fabrics are now popular among major brands and top designers.

Our aim is to lead students to study in teams through a crowdfunding plan competition. We emphasize and attach importance to the learning process and crowdfunding plans, rather than the actual crowdfunding. A key challenge in the development of crowdfunding is to provide students with meaningful opportunities to raise funds and allow them to learn how to become resilient and capable by carrying out this work. However, as a by-product of students’ efforts, the lecturer may be able to see a small number of feasible “products and crowdfunding plans.” Teachers can encourage and guide these student teams to continue with their crowdfunding process.

## Conclusion

This article focuses on encouraging and educating student teams about the process of creating a matched crowdfunding plan for market demands and products. A team must show market and economic sustainability for their plan to have the opportunity to raise funds successfully from crowdfunding platforms. Hopefully, every student taking this course will be able to achieve this goal. Furthermore, this study can influence our methods of teaching. OBE is akin to learning through actions and producing outcomes. Action-oriented learning is a learning theory which entrepreneurs use to learn by reflecting on the actions they have taken when solving practical problems. Therefore, on the basis of OBE, we collated relevant literature concerning crowdfunding as potential teaching content, thereby developing a course covering the material on this topic.

This course’s development makes four contributions: (1) this article integrates Jolly’s model and OBE, and puts forward the process of crowdfunding competition. The former values the commercialization process while the latter focuses on results. We divided the crowdfunding program into three parts, allowing participants to make achievements gradually by running fast using small steps. This allows learners to learn from doing, observe peers and reflect, and develop commercial products and crowdfunding plans; (2) by combining research and teaching, important concepts are extracted from the relevant literature on successful crowdfunding. Topics discussed by scholars are then taught so that students can understand the key factors affecting the success of crowdfunding. The gap between management research and teaching has also received attention from scholars (Burke and Rau, 2010). Thus, this method provides a new path for future course design; (3) differing from other entrepreneurship or crowdfunding courses, this course combines the two so as to induce students to learn from the practical aspects of product development and crowdfunding; and (4) in most courses, students are divided into groups from within the course to carry out activities; this course, however, encourages learners to invite students external to the course in order to form teams. The instructors are not limited to the course lecturer: teachers in the fields related to the entrepreneurial projects can be invited. This crowdfunding course is built as a platform for multidisciplinary interactions, thereby enhancing interdisciplinary thinking (Kazakeviciute et al., 2016).

The crowdfunding plan competition is not only an integral part of product commercialization, but also an educational mechanism concerning the development process of fundraising. It can, thus, build a bridge between “products, crowdfunding plan competition, and entrepreneurship.” The most important factors regarding this process are innovation and creativity. Throughout the stages of imagination, incubation, planning, display, promotion, and so on, students are required to draw upon their innovation and creativity.

In the teaching goals, the teaching philosophy methods can be changed according to the teachers’ plans. For example, if teachers feel that there is not enough class time, they can use autonomous learning to supplement students’ development for the purposes of the course. At present, assessment is conducted by teachers and students. The lecturer can introduce experts from the platform, consumers, and business circles to conduct assessments. This course does not require the student teams to actually raise funds. The main reason being that it is difficult to develop good products within one semester. If conditions permit, teachers can set the requirements in the course arrangement period, but the teaching progress may need to be rescheduled.

The teaching content is mainly derived from the extant body of literature concerning crowdfunding. For the lecturer, this is a dynamic process. With the development of research, teachers can add or remove some content, or can designate some content as autonomous learning content. We have also tried our best to encourage students to seek out team members outside of the course’s enrolled class members so as to form interdisciplinary teams. This can assist and encourage students to learn how to integrate experience, skills and knowledge, and prepare for starting new businesses. Since we encourage students to form diversified teams, the cross-functional teaching team can broaden students’ horizons. Students will be exposed to different viewpoints on the process of crowdfunding, and should deal with every problem in this process from multiple perspectives.

The arrangement of the number of teaching weeks can be adjusted according to teachers’ plans. It is suggested that the lecturer can add or remove autonomous learning sections to adjust the number of teaching weeks. If previous courses already included entrepreneurship and new product development, and the student teams already have their product conceptions, this course could just focus on how to develop a crowdfunding plan. Crowdfunding course can also be developed into a school year-long program, with the first semester focusing on product development and the second semester focusing on the development of crowdfunding plans. Throughout the course, students can raise funds *via* crowdfunding platforms and obtain the first-hand feedback on their products and services from actual real-world fundraising situations.

This course has not been taught yet, and still needs the input and verification of the authors and additional teachers. The first module and competition process have been taught by the second author and some students taking the course achieved good results in many entrepreneurship competitions. Teaching knowledge and creating knowledge are complementary activities (Burke and Rau, 2010). Therefore, we adopt a new viewpoint, sorting out the themes of the literature on crowdfunding as the potential teaching content. The proposed course design method can be extended and applied to other present and future education fields (Wu and Chen, 2021b).

## Data Availability Statement

The original contributions presented in this study are included in the article/supplementary material, further inquiries can be directed to the corresponding author/s.

## Author Contributions

YW: conceptualization, and review and editing. C-HY: writing. Both authors contributed to the article and approved the submitted version.

## Conflict of Interest

The authors declare that the research was conducted in the absence of any commercial or financial relationships that could be construed as a potential conflict of interest.

## Publisher’s Note

All claims expressed in this article are solely those of the authors and do not necessarily represent those of their affiliated organizations, or those of the publisher, the editors and the reviewers. Any product that may be evaluated in this article, or claim that may be made by its manufacturer, is not guaranteed or endorsed by the publisher.
